# Evaluation of Oropharyngeal pH-Monitoring in the Assessment of Laryngopharyngeal Reflux

**DOI:** 10.3390/jcm10112409

**Published:** 2021-05-29

**Authors:** Lukas Horvath, Patricia Hagmann, Emanuel Burri, Marcel Kraft

**Affiliations:** 1 Department of Otorhinolaryngology, Head and Neck Surgery, University Hospital of Basel, 4031 Basel, Switzerland; 2 Department of Gastroenterology and Hepatology, University Medical Clinic, Kantonsspital Baselland, 4410 Liestal, Switzerland; patricia.hagmann@gmx.ch (P.H.); emanuel.burri@ksbl.ch (E.B.); 3 HNO-Zentrum beider Basel, 4141 Münchenstein, Switzerland; marcel.kraft@unibas.ch

**Keywords:** extraesophageal reflux, Dx-pH measuring system, pH-metry, pH testing, reflux disease

## Abstract

Background: Laryngopharyngeal reflux (LPR) is a prevalent disorder. The aim of the present retrospective cohort study was to evaluate oropharyngeal pH-monitoring using a novel scoring system for LPR. Methods: In a total of 180 consecutive patients with possible LPR, reflux symptom index (RSI), reflux finding score (RFS), oropharyngeal pH-monitoring and transnasal esophagoscopy were carried out for further investigation. Results: In our series, 99 (55%) patients had severe LPR, 29 (16%) cases presented with moderate and 23 (13%) with mild severity, 9 (5%) subjects revealed neutral values, and 7 (4%) individuals were alkaline, while 13 (7%) patients had no LPR. In detecting LPR, the sensitivity, specificity and accuracy of oropharyngeal pH-monitoring was 95%, 93% and 94%, respectively. Conclusion: Oropharyngeal pH-monitoring is a reliable tool in the assessment of LPR, but the pH graphs have to be precisely analyzed and interpreted in context with other validated diagnostic tests.

## 1. Introduction

Laryngopharyngeal reflux (LPR) was recognized as an independent clinical entity in 2002 by the American Academy of Otolaryngology–Head and Neck Surgery [1]. It represents a laryngopharyngeal manifestation of refluxed gas and/or fluids from the gastrointestinal tract consisting of various pH values, causing local symptoms and disease. In such a manner, LPR should neither be considered a lesser version of, nor a manifestation of, gastroesophageal reflux (GER). Nevertheless, GER and LPR can also occur simultaneously [1]. Laryngopharyngeal reflux occurs mostly during the daytime in the upright position and shows a short-term reflux exposure to the throat [1]. Its primary defect is considered an upper and/or lower esophageal sphincter dysfunction [2]. The refluxate mainly comprises acids, bile salts or pepsin causing local irritation of the exposed mucosa [3]. Symptoms are generally nonspecific, such as hoarseness, chronic cough, globus sensation, acid regurgitation, esophageal dysmotility and dysphagia [4]. In contrast to GER, a universal norm for LPR diagnosis has been lacking thus far. Though LPR is a common disease among the general population, it is often misdiagnosed as GER and, therefore, treated as such, often without success [5,6]. Additionally, the majority of general practitioners and otolaryngologists do not consider themselves well versed in managing LPR, leading to differing diagnostic and treatment approaches [7]. Thus, the reflux symptom index (RSI), the reflux finding score (RFS) and/or a positive response to a trial of proton pump inhibitor (PPI) therapy have been applied as diagnostic criteria. These patients are often directly referred to a gastroenterologist for esophagogastroduodenoscopy, and there seems to be no unified diagnostic approach for LPR. Many clinicians use only one diagnostic method at a time [7]. However, there is no single test capable of diagnosing LPR alone, while oropharyngeal pH-monitoring for detecting LPR is also a matter of debate in the current literature [8,9]. Meanwhile, standard values for oropharyngeal pH-monitoring have been established through healthy subjects, and a positive Ryan Score has been considered synonymous for the presence of LPR [10,11,12]. Recently, we proposed a novel scoring system for LPR, which combines four validated diagnostic methods, namely RSI, RFS, oropharyngeal pH-metry and transnasal esophagoscopy [13]. As the Horvath Score simultaneously comprises sensitive and specific tests, as well as subjective and objective criteria, it is associated with a much higher probability to identify true LPR than any other method used in the past. The aim of the present study was to evaluate oropharyngeal pH-monitoring in the assessment of LPR, identifying possible pitfalls and developing strategies to avoid them, while the Horvath Score served as the gold standard for LPR diagnosis.

## 2. Materials and Methods

A total of 180 consecutive patients with possible LPR were included in the present study using the same inclusion criteria as published before [13]. Subsequently, RSI and RFS were gathered [14,15]. Oropharyngeal pH-metry and transnasal esophagoscopy were then carried out for further investigation and classified according to the Horvath Score as previously described [13]. The latter was used as the gold standard for LPR diagnosis. In this regard, our study protocol was compliant with the ethical standards of the Declaration of Helsinki and approved by the local ethics committee (EKNZ 2019-01650).

### 2.1. Oropharyngeal pH-Monitoring (PHM)

The Restech probe (Respiratory Technology Corporation, San Diego, CA) was used for oropharyngeal pH-monitoring. First, the probe was calibrated in buffer solutions according to the manufacturer’s instructions. Following topical intranasal anesthesia with lidocaine spray, a drop-shaped pH sensor, based on antimony technology and housed at the tip of the probe, was introduced transnasally through the wider nasal cavity into the oropharynx. With the help of a data logger, the sensor registered liquid and aerosolized refluxate charting pH values without contact, twice per second for 24 h. The small 2 mm probe was positioned below the uvula under visual guidance, while a blinking LED light at the tip of the probe helped with correct positioning [16]. Once placed accurately, the catheter running to the data logger was affixed on the cheek with tape to avoid dislocation. The logger was attached to the collar as a badge and transmitted data wirelessly to the recorder, which in turn could be carried as a purse [17]. The patient was instructed to log mealtimes (incl. beverages, except for pure water), body position (e.g., upright or supine) and individual LPR symptoms (e.g., chronic cough, globus sensation, throat clearing, heartburn) by pressing a button. The small probe was generally well tolerated, and as an outpatient measuring method, patients were asked to go about their daily routine to represent everyday life. All patients with PPI therapy suspended its use at least 8 days before PHM.

**Figure 1:**
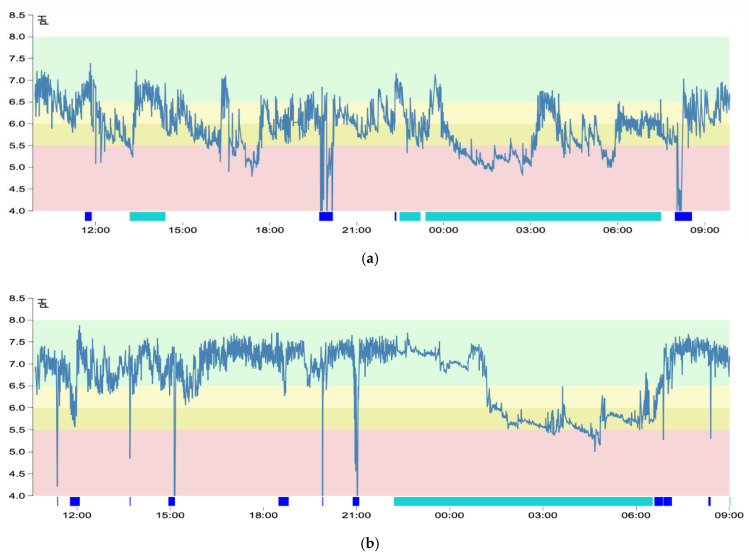

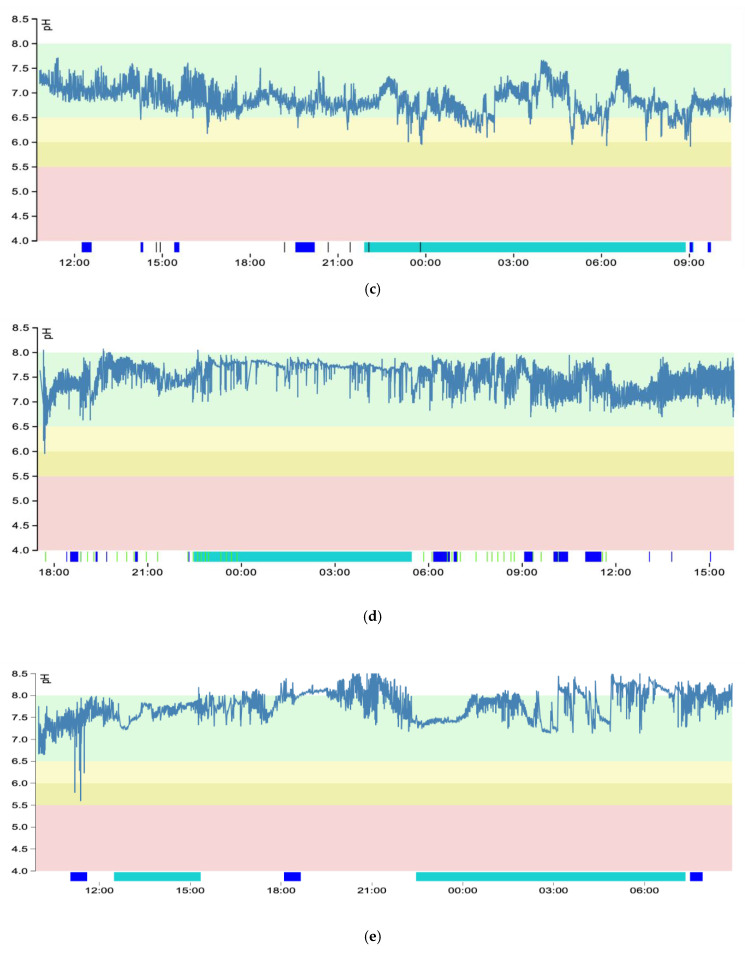

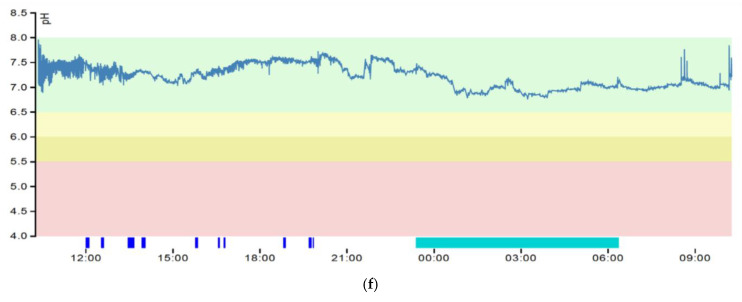
(**a**) Severe laryngopharyngeal reflux—blue bar = mealtimes; green bar = supine position. (**b**) Moderate laryngopharyngeal reflux—blue bar = mealtimes; green bar = supine position. (**c**) Mild laryngopharyngeal reflux—blue bar = mealtimes; green bar = supine position. (**d**) Neutral laryngopharyngeal reflux—blue bar = mealtimes; green bar = supine position. (**e**) Alkaline laryngopharyngeal reflux—blue bar = mealtimes; green bar = supine position. (**f**) No laryngopharyngeal reflux—blue bar = mealtimes; green bar = supine position.

In the Restech graph, pH values are shown over 24 h in upright and supine positions, whereas meals and logged symptoms are indicated below the graph. The pH values during mealtimes are removed from calculation of the Ryan Score, whereas pH values during symptoms are used for correlation [18]. The severity of extraesophageal reflux is assessed with the help of the Ryan Score. The latter comprises the total time of pH < 5.5 in the upright position or pH < 5.0 in the supine position, as well as the number and length of each reflux period. A positive Ryan Score means severe reflux, whereby higher values are associated with a greater acid exposure in the throat [10]. However, a negative Ryan Score does not exclude moderate, mild, neutral or alkaline LPR, as well as no reflux. Therefore, the type and severity of LPR is finally defined by wave amplitudes and pH levels and not the Ryan Score alone. A graph running below pH 5.5 in the upright position or below pH 5.0 in the supine position, respectively, was considered severe LPR (Figure 1a). A graph between pH 5.5 and 6.0 in the upright position or between pH 5.0 and 5.5 in the supine position, respectively, was considered as moderate LPR (Figure 1b), while pH 6.0–6.5 signified mild LPR in the upright or pH 5.5–6.0 in the supine position (Figure 1c). A neutral spectrum was between pH 6.5 and 8.0 upright or pH 6.0–8.0 supine (Figure 1d), and all values above pH 8.0 were considered alkaline (Figure 1e). The differentiation between neutral LPR and no reflux can only be made by amplitude width (Figure 1f). Wave amplitudes within a given spectrum should be critically assessed because single outliners and slight nocturnal drops in pH levels may well be artifacts, whereas consistent wide amplitudes signify active reflux. During the time course of the pH graph composed of wide amplitudes, the lowest or highest consistent pH level determines the severity of LPR. All patients were thoroughly instructed in the handling of the device and were additionally requested to record events in a written symptom diary, which could be used the following day to correlate with logged events on the device. In case of a preexisting PPI therapy, the latter was suspended for at least one week prior to testing, as proposed in the literature [19]. With the help of the Restech Dataview v4 Software, each graph was analyzed for wave amplitudes, logged symptoms, pH levels and mean pH-values, taking account of mealtimes and body position.

### 2.2. Horvath Score

The Horvath Score classifies LPR into 5 degrees of severity (severe, moderate, mild, neutral, alkaline) or excludes extraesophageal reflux. This scoring system comprises 4 validated diagnostic methods (RSI, RFS, PHM, TNE) consisting of subjective and objective criteria for LPR, providing 1 point each when pathological findings were noted, or 0 points when normal findings were present, as previously depicted [13].

### 2.3. Statistics

The calculation of sensitivity, specificity, accuracy, and positive and negative predictive values in detecting LPR was carried out separately for each diagnostic method. Oropharyngeal pH-metry and RSI >13 in severe and nonsevere LPR were regarded as positive and negative in nonexistent reflux. A Reflux Finding Score >7 was considered as positive in severe and moderate LPR and negative in all other cases. Transnasal esophagoscopy was rated positive if characteristic reflux findings were seen (e.g., sphincter insufficiency, hiatal hernia, gaping cardia, visible reflux, peptic esophagitis, Barrett esophagus, ectopic gastric mucosa), whereas ordinary findings were regarded as negative. Fisher’s exact test was applied for statistical analysis (Fisher’s exact test add-in in MS Excel). A *p*-value of <0.05 was regarded statistically significant, while *p*-values of <0.01 were considered highly significant.

## 3. Results

### 3.1. Clinical Data

Our series consisted of 93 male (52%) and 87 female subjects (48%). The mean age at diagnosis was 63 years (range, 21–93 years). The results of each diagnostic test are depicted in Table 1, Table 2, Table 3 and Table 4.

### 3.2. Oropharyngeal pH-Monitoring

Initially, 100 (55%) patients showed a positive Ryan Score implicating severe LPR, and 28 (16%) subjects displayed a moderate severity. Additionally, 23 (13%) cases presented with mild LPR, 9 (5%) individuals revealed neutral values, 7 (4%) subjects were alkaline, and 13 (7%) patients had no LPR (Table 2).

According to the Horvath Score, 6 false-positive (5 moderate LPR and 1 mild LPR instead of severe LPR) and 5 false-negative results (4 severe LPR instead of moderate LPR and 1 severe LPR instead of mild LPR) for severe LPR were found in our cohort (Table 3).

Finally, 99 (55%) patients had severe LPR showing a Horvath Score of 4–5, 29 (16%) cases presented with moderate and 23 (13%) with mild severity with a Horvath Score between 2 and 3, 9 (5%) subjects revealed neutral values, and 7 (4%) individuals were alkaline, also reaching a Horvath Score between 2 and 3. Furthermore, 13 (7%) patients had no LPR presenting with a Horvath Score of 0–1 (Table 2).

In detecting LPR, oropharyngeal pH-monitoring showed a significantly higher sensitivity (95% vs. 78% and 52%) and accuracy (94% vs. 77% and 63%) than RSI and RFS, but a similar sensitivity (95% vs. 97%) and accuracy (94% vs. 96%), compared to transnasal esophagoscopy. In contrast, the specificity (93% vs. 54%) of oropharyngeal pH-monitoring was significantly higher than that of RSI and remained similar (93% vs. 94% and 88%) in comparison to RFS and transnasal esophagoscopy (Table 4).

### 3.3. pH Graphs

For educational purposes, different graphs from oropharyngeal pH-monitoring are presented in Figure 1a–f. The Restech graph is read from left to right and represents the pH levels (*y*-axis) over a timeline of approximately 24 h (*x*-axis). The background colors mark the different types and severity of LPR (i.e., red = severe acid LPR, dark yellow = moderate acid LPR, bright yellow = mild acid LPR, green = neutral or no LPR). The blue bar below the graph marks mealtimes, which were omitted from the calculation. The green bar marks a supine position, and the occasional thin lines represent subjective symptoms (e.g., cough, heartburn) logged by the patient.

Severe laryngopharyngeal reflux showed wide wave amplitudes reaching below the threshold of pH 5.5 in upright and 5.0 in supine position during meals, as well as independent of mealtimes (Figure 1a).

Moderate laryngopharyngeal reflux was composed of wide wave amplitudes reaching an area between pH 5.5 and 6.0 in upright and pH 5.0 and 5.5 in supine position. All deep drops in pH levels were due to mealtimes and were correctly logged; thus, they were excluded from calculation. During supine position, pH levels dropped in a smooth manner, in this case to moderate levels. This phenomenon is known as the nocturnal drop and drift (Figure 1b).

Mild laryngopharyngeal reflux illustrated wide wave amplitudes within pH 6.0 and 6.5 upright and pH 5.5–6.0 in supine position. Deep drops in pH levels were not recognized during mealtimes (Figure 1c).

Neutral laryngopharyngeal reflux presented with wide wave amplitudes within pH 6.5 and 8.0, which is associated with a pathologic reflux symptom index and pathologic findings in transnasal esophagoscopy. Deep drops in pH levels were not recognized during mealtimes (Figure 1d).

Alkaline laryngopharyngeal reflux had wide wave amplitudes reaching above the threshold of pH 8.0 in the upright and supine positions. The deep drops in pH levels below 6.5 were associated with mealtimes and were, therefore, excluded from calculation (Figure 1e).

No laryngopharyngeal reflux displayed tight wave amplitudes with pH in the normal range within pH 6.5 and 8.0. No reflux events occurred, even during mealtimes (Figure 1f).

## 4. Discussion

This is the first study in the literature that critically evaluated oropharyngeal pH-monitoring in the assessment of LPR. Furthermore, this paper specified how to correctly analyze and interpret such pH graphs. The gold standard for diagnosing GER is esophagogastroduodenoscopy and traditional impedance pH-testing; however, a universal approach to assess LPR is not yet defined [20]. As traditional impedance pH-metry has failed in the throat due to false-positive and -negative results, the gold standard for GER is not suitable to detect LPR. Combined hypopharyngeal-esophageal multichannel intraluminal impedance pH-testing might be a better option and a potential alternative to oropharyngeal pH-monitoring, but this needs further evaluation [19,21].

Though oropharyngeal pH-monitoring is widely used for LPR detection, it is not generally accepted as a gold standard for diagnosing extraesophageal reflux. One reason might be that a few studies evaluating oropharyngeal pH-monitoring were using this method as reference itself for LPR diagnosis, which is scientifically flawed [22,23]. In such a manner, the lack of acceptance seems to be partly understandable as statistical errors exist in every diagnostic method, including oropharyngeal pH-monitoring. However, the downward-facing drop-shaped antimony tip of the Restech probe avoids being covered by mucus or food, prevents it from drying out, is capable of detecting both liquid and gaseous droplets in the pharynx and does not require contact with fluid or tissue for electrical continuity. Essentially, this would make the Restech probe an ideal instrument for the detection of extraesophageal reflux, but there is still a need to further investigate the diagnostic value of this method, which was the main goal of this study [18,24].

In our study, we evaluated oropharyngeal pH-monitoring using a novel scoring system for LPR and observed a few false positive and negative results. Six patients were rated to have severe LPR by showing a positive Ryan Score. A pH graph with wide amplitudes dropping below the threshold, cohesive logged subjective symptoms and a positive Ryan Score were generally found in severe LPR (e.g., Figure 1a). However, those particular pH graphs revealed artifacts, which needed to be deleted from calculation, resulting in a negative Ryan Score and a typical graph for moderate severity (pH 5.5–6.0). An occasionally occurring artifact was that patients forgot to log mealtimes, which led to massive drops in the pH level during eating or drinking (e.g., Figure 1e). To overcome this issue, patients were asked to note their mealtimes, body position and symptoms on a separate hand-written log sheet. The following day, the graph was analyzed together with the patient, allowing for a discussion regarding extraordinary events along the graph and to correct the aforementioned artifacts. Nevertheless, acid exposure during mealtimes needs to be observed and may be important to consider in lifestyle modifications and/or treatment recommendations. Additionally, we noted that some patients incorrectly logged their body position, especially when standing up at night or in the morning after waking. Due to slight nocturnal drops in acidity, the threshold for severe LPR is lowered to pH 5.0 in the supine position, while in an upright position the threshold is located at pH 5.5. The distinction between the upright and supine position is of great importance due to different cut-off points. Failing to correctly log the latter leads to a false calculation of the Ryan Score and improper display of the pH graph. Furthermore, a potential artifact can occur by placing the recorder too far away, thus causing a loss of connection between the transmitter and the recorder. This is characterized on the graph as a straight line from the last transmitted data point to the next received, and no calculation is made during this period. One patient in our series was rated to have moderate reflux, but in context with the other diagnostic methods, he instead had severe LPR. Thus, despite a negative Ryan Score, this finding ultimately made him a candidate for surgical therapy as he did not respond sufficiently to lifestyle modifications and pharmacotherapy. Two patients in our study had neutral LPR, showing wide amplitudes in the recorded graph with values between pH 6.5 and 8.0. These patients also had pathologic findings in RSI and transnasal esophagoscopy. Yet it is challenging to discriminate neutral from nonexisting LPR, which is only possible in context with RSI, RFS and transnasal esophagoscopy. Therefore, the pH graph should not be the conclusive determinant for final LPR diagnosis, but the latter needs to be interpreted concurrently with other validated diagnostic tests. In such a manner, we found patients with neutral or alkaline LPR occasionally achieving a Horvath Score of 4 indicating severe reflux disease. These cases would probably also benefit from antireflux surgery due to the lack of an adequate medical therapy.

There might be a few limitations to our study. A retrospective design is generally not an ideal concept. In such a manner, a prospective approach would be better to evaluate oropharyngeal pH-monitoring. Additionally, there is a selection bias in our cohort, as only patients with signs and symptoms suspicious for LPR were chosen for further investigation. However, including healthy individuals without any symptoms to be tested by all four diagnostic methods is ethically difficult to justify. Furthermore, patients with GER and gastritis can present with similar symptoms of RSI, yet cannot be identified by oropharyngeal pH-monitoring. Therefore, if patients test negative in oropharyngeal pH-metry, they may still have GER or gastritis instead of LPR. At present, nothing is known about the internal consistency and interrater reproducibility of PHM. Hence, more studies are needed to externally validate this method.

Many studies exploring the utility of oropharyngeal pH-monitoring in LPR diagnosis considered the Ryan Score as a basis for LPR detection [24,25]. Though a positive Ryan Score implies the presence of LPR, extraesophageal reflux cannot be excluded on the basis of a negative Ryan Score. If the patient’s values for the upright and/or supine position surpass the cut-off points, the Ryan Score becomes positive, and a positive Ryan Score means a patient has “severe” reflux. Accordingly, the Ryan Score has initially been developed with high specificity for severe LPR, whereas a negative Ryan Score can still be indicative of nonsevere LPR [10]. Thus, a negative Ryan Score is insufficient to exclude LPR. In such a manner, the Restech graph needs to be examined precisely, observing pH levels, width of wave amplitudes, mean pH value, logged symptom correlation and possible artifacts within the graph. Additionally, RSI is needed to estimate the patient’s discomfort, RFS to assess the mucosal changes in the larynx and transnasal esophagoscopy to display the underlying pathology.

## 5. Conclusions

Laryngopharyngeal reflux (LPR) is a prevalent disorder in the general population, and a novel scoring system has recently been proposed for its diagnosis. Oropharyngeal pH-monitoring alone, or in combination with other diagnostic tests, is frequently applied in an LPR diagnosis but is controversial and bears numerous pitfalls. Being aware of the latter, it can be concluded that oropharyngeal pH-monitoring is a reliable tool in the assessment of LPR. However, diagnosing LPR or excluding LPR cannot be solely made by PHM alone, rather it needs to be interpreted in synopsis with other validated diagnostic methods such as RSI, RFS and TNE. Additionally, our study makes the reader aware of the value of PHM and the pitfalls encountered in its interpretation. Describing how to read and interpret Restech graphs makes the present study not only valuable for other research groups, but also for the clinician at the bedside.

## Figures and Tables

**Table 1:** Study results for each method in detecting laryngopharyngeal reflux according to the Horvath Score (*n* = 180).

Horvath Score	Severity	Pathologic RSI	Pathologic RFS	Pathologic PHM	Positive Ryan	Pathologic TNE	Total
4–5	Severe LPR	79 (80%)	57 (58%)	99 (100%)	94 (95%)	97 (98%)	99 (55%)
2–3	Nonsevere LPR	52 (76%)	15 (22%)	65 (96%)	6 (9%)	62 (91%)	68 (38%)
	Moderate	20 (69%)	7 (24%)	27 (93%)	5 (17%)	25 (86%)	29 (16%)
	Mild	16 (70%)	3 (13%)	23 (100%)	1 (4%)	21 (91%)	23 (13%)
	Neutral	9 (100%)	3 (33%)	8 (89%)	0 (0%)	9 (100%)	9 (5%)
	Alkaline	7 (100%)	2 (29%)	7 (100%)	0 (0%)	7 (100%)	7 (4%)
0–1	No LPR	6 (46%)	0 (0%)	0 (0%)	0 (0%)	2 (15%)	13 (7%)

Abbreviations: LPR, laryngopharyngeal reflux; RSI, Reflux Symptom Index; RFS, Reflux Finding Score; PHM, oropharyngeal pH-monitoring; Ryan, Ryan Score; TNE, transnasal esophagoscopy.

**Table 2:** Results of oropharyngeal pH-monitoring before and after application of Horvath Score (*n* = 180).

PHM	Severe LPR	Moderate LPR	Mild LPR	Neutral LPR	Alkaline LPR	No LPR
Initial	100 (55%)	28 (16%)	23 (13%)	9 (5%)	7 (4%)	13 (7%)
Final	99 (55%)	29 (16%)	23 (13%)	9 (5%)	7 (4%)	13 (7%)

Abbreviations: LPR, laryngopharyngeal reflux; PHM, oropharyngeal pH-monitoring; Initial, initial interpretation of Restech graph; Final, final results of Restech graph after application of Horvath Score.

**Table 3:** Detection of laryngopharyngeal reflux (*n* = 180).

Method	True Positive	False Positive	False Negative	True Negative	Total
RSI	131	6	36	7	180
RFS	69	3	64	44	180
PHM	94	6	5	75	180
TNE	159	2	5	14	180

Abbreviations: RSI, Reflux Symptom Index; RFS, Reflux Finding Score; PHM, oropharyngeal pH-monitoring; TNE, transnasal esophagoscopy.

**Table 4:** Detection of laryngopharyngeal reflux (*n* = 180).

Method	Sensitivity	Specificity	Accuracy	Pos. Pred. Value	Neg. Pred. Value
RSI	78%^†^ (*p* = 0.000)	54%^†^ (*p* = 0.001)	77%^†^ (*p* = 0.000)	96% (*p* = 0.392)	16% ^†^ (*p* = 0.000)
RFS	52%^†^ (*p* = 0.000)	94% (*p* = 0.566)	63%^†^ (*p* = 0.000)	96% (*p* = 0.434)	41% ^†^ (*p* = 0.000)
PHM	95%	93%	94%	94%	94%
TNE	97% (*p* = 0.306)	88% (*p* = 0.492)	96% (*p* = 0.235)	99%* (*p* = 0.038)	74% * (*p* = 0.021)

Abbreviations: RSI, Reflux Symptom Index; RFS, Reflux Finding Score; PHM, oropharyngeal pH-monitoring; TNE, transnasal esophagoscopy. Note: Pathologic findings are classified as positive and normal findings as negative for the calculation of sensitivity, specificity, accuracy, and positive and negative predictive values. * Statistical significance (*p* < 0.05); ^†^ high statistical significance (*p* < 0.01).

## Data Availability

The data presented in this study are available on request from the corresponding author. The data are not publicly available due to privacy or ethical restrictions.
